# Early Access to Oral Antivirals in High-Risk Outpatients: Good Weapons to Fight COVID-19

**DOI:** 10.3390/v14112514

**Published:** 2022-11-14

**Authors:** Giuseppe Bruno, Massimo Giotta, Serena Perelli, Giuseppina De Vita, Nicola Bartolomeo, Giovanni Battista Buccoliero

**Affiliations:** 1 Infectious Diseases Unit, San Giuseppe Moscati Hospital, Azienda Sanitaria Locale Taranto, 74121 Taranto, Italy; 2 Complex Unit of Statistics and Epidemiology, Azienda Sanitaria Locale Taranto, 74121 Taranto, Italy; 3 Interdisciplinary Department of Medicine, University of Bari Aldo Moro, 70121 Bari, Italy

**Keywords:** COVID-19, antivirals, SARS-CoV-2, Molnupiravir, Nirmatrelvir

## Abstract

Introduction: Molnupiravir and Nirmatrelvir/r (NMV-r) have been proven to reduce severe Coronavirus Disease 2019 (COVID-19) in unvaccinated high-risk individuals. Data regarding their impact in fully vaccinated vulnerable subjects with mild-to-moderate COVID-19 are still limited, particularly in the era of Omicron and sub-variants. Methods: Our retrospective study aimed to compare the safety profile and effectiveness of the two antivirals in all consecutive high-risk outpatients between 11 January and 10 July 2022. A logistic regression model was carried out to assess factors associated with the composite outcome defined as all-cause hospitalization and/or death at 30 days. Results: A total of 719 individuals were included: 554 (77%) received Molnupiravir, whereas 165 (23%) were NMV-r users. Overall, 43 all-cause hospitalizations (5.9%) and 13 (1.8%) deaths were observed at 30 days. A composite outcome occurred in 47 (6.5%) individuals. At multivariate analysis, male sex [OR 3.785; *p* = 0.0021], age ≥ 75 [OR 2.647; *p* = 0.0124], moderate illness [OR 16.75; *p* < 0.001], and treatment discontinuation after medical decision [OR 8.148; *p* = 0.0123] remained independently associated with the composite outcome. Conclusions: No differences between the two antivirals were observed. In this real-life setting, the early use of both of the oral antivirals helped limit composite outcome at 30 days among subjects who were at high risk of disease progression.

## 1. Introduction

Since its rapid spread starting in December 2019, as of 23 October 2022, the COVID-19 pandemic caused over 624 million confirmed cases and over 6.5 million deaths have been reported globally [1]. The severe acute respiratory syndrome Coronavirus-2 (SARS-CoV-2) after its entry mainly through the respiratory tract is capable of inducing a vehement inflammatory response, which is considered the hallmark of the infection. In fact, its various structural and non-structural proteins can directly or indirectly stimulate the uncontrolled activation of harmful inflammatory pathways causing cytokine storm, tissue damage, increased pulmonary edema, acute respiratory distress syndrome (ARDS) and mortality [2].

COVID-19 still represents a threat to the healthcare systems and has significant social implications in terms of morbidity, absence due to illness in the workplace, and direct and indirect costs [3]. Numerous therapeutic strategies have been developed to prevent the infection (vaccines and monoclonal antibodies) and to slow down the progression to severe COVID-19 (anti-inflammatory molecules, steroids, heparin, and antivirals) [4].

Moreover, the dominant variants of SARS-CoV-2 are constantly evolving. Data regarding variants of the SARS-CoV-2 in Italy are periodically updated by the National healthcare Institute [5]. As of January 2022, Omicron and subvariants constituted over 90% of all SARS-Cov2 infections in Italy.

In the current scenario, circulating Omicron variants (BA.2, BA.4, and BA.5) have significantly reduced susceptibility to several monoclonal antibodies (mAbs), including casirivimab–imdevimab, bamlanivimab–etesevimab, and sotrovimab [6,7]. The combination tixagevimab–cilgavimab, initially authorized only in pre-exposure prophylaxis, appears to be still active toward BA.2 and, albeit to a lesser extent, toward BA.1, BA.1.1, and the recent BA.4 and BA.5 [6,7,8].

Importantly, anti-SARS-CoV-2 vaccination still represents the main mean for limiting the spread of infection and reducing the risk of worse outcomes. However, the effectiveness of vaccines tends to decrease over time, due, on the one hand, to the ability of SARS-CoV-2 to modify itself [9], and on the other hand, to the impaired immune system, particularly among fragile and high-risk individuals [10]. At the end of December 2021, European Medicines Agency (EMA) authorized the emergency use of two antivirals against SARS-CoV-2, Molnupiravir, and Nirmatrelvir/r (NMV-r) to prevent severe illness in high-risk individuals who are not hospitalized for COVID-19 and with no need for supplemental oxygen [11]. Molnupiravir is a prodrug of beta-d-N4-hydroxyxcytidine acting as an oral inhibitor of RNA-dependent RNA polymerase that can increase the viral RNA mutations, thus impairing the replication of SARS-COV2 [12]. NMV-r is an orally administered antiviral agent targeting the SARS-CoV-2 3-chymotrypsin-like cysteine protease enzyme (Mpro) that is essential in the viral replication cycle [13]. Despite the fact that their efficacy has been demonstrated in randomized trials [14,15], real-life data regarding their impact on fully vaccinated vulnerable subjects with mild-to-moderate COVID-19 are still limited, particularly in the era of Omicron and subvariants. Hence, we aim to assess the safety and effectiveness of the two antivirals in terms of the composite outcome defined as all-cause hospitalization and/or death at 30 days.

## 2. Materials and Methods

### 2.1. Clinical Setting

Our hospital, “San Giuseppe Moscati” of Taranto, is a COVID-19 referral hub in Apulia, Southern Italy. We included in this retrospective study all consecutive individuals with confirmed COVID-19 and mild-to-moderate illness who received an oral antiviral prescription in Taranto and its Province between 11 January and 10 July 2022. Sociodemographic, as well as clinical, data were collected in a dedicated database that included comorbidities, daily taken drugs, time from the onset of symptoms to antiviral prescription, date of COVID-19 vaccinations, side effects in the course of treatment, and clinical outcomes at 30 days after the treatment initiation.

General Practitioners and Special Units for Continuity of Care (USCA) identified high-risk patients with COVID-19 and sent a formal request for eligibility for antiviral therapy. We assessed each patient according to the Italian Medicine Agency (AIFA) criteria [16]. Therefore, antiviral therapy was selected after carefully evaluating drug–drug interactions by consulting a dedicated website https://www.covid19-druginteractions.org (accessed on 3 November 2022) [17]. Before starting treatment, all subjects received an information form on the prescribed antiviral and signed informed consent. In addition, women with childbearing potential were advised to use an effective method of contraception, which necessarily includes a barrier method, for the whole duration of the treatment and at least four days after the end of Molnupiravir treatment. Male partners of women with childbearing potential were required to ensure contraception for the total treatment duration and at least three months after the end of Molnupiravir treatment.

In the presence of clinical signs of worsening (persistent fever, onset of breathlessness, reduced oxygen saturation, etc.), patients themselves or their caregivers were asked to contact the GPs who activated the Special Units for Continuity of Care (USCA) or, in severe cases, the Italian emergency telephone number, 118. Alternatively, our team was contacted directly and provided clinical suggestions.

### 2.2. Criteria Inclusion

Criteria inclusion of the study were (1) age ≥ 18 years, (2) COVID-19 confirmed by antigenic or molecular swab, (3) subjects who have taken at least one dose of antiviral, (4) onset of symptoms within five days, and (5) at least one of the following comorbidities: obesity (body mass index ≥ 30); diabetes mellitus with organ damage or HBa1c > 7.5%; chronic renal failure; chronic respiratory diseases; severe cardiovascular disease; primary or secondary immunodeficiency; malignancies; neurological disease; and age ≥ 65.

### 2.3. Criteria of Exclusion

The criteria for exclusion were (1) pregnancy, (2) patients who refused to take the therapy, (3) severe illness requiring oxygen support and/or hospitalization due to COVID-19, (4) patients already hospitalized, (5) severe liver impairment, and (6) severe renal impairment (eGFR < 30 mL/min/1.73 m^2^).

Mild-to-moderate illness was defined as reported in the COVID-19 Treatment Guidelines Panel [18]. Both antivirals were administered for five days according to the dosage recommended by the manufacturers [16].

### 2.4. Endpoints

The first endpoint was to assess of the two antivirals in terms of the composite outcome defined as all-cause hospitalization and/or death at 30 days, as reported. The second endpoint was to compare their safety profile. Third, we aimed to identify factors associated with the composite outcome.

### 2.5. Statistics

Quantitative data were shown as means and standard deviation (SD) if normally distributed, and as median and interquartile range (IQR) if assumption of normality was not acceptable. Shapiro–Wilk’s statistics was used to test normality. Differences in continuous variables between two groups defined by the primary (composite outcome) or secondary (antiviral therapy) endpoint were compared by using Student’s *t*-test for normally distributed parameters, or the nonparametric Mann–Whitney U test otherwise. Categorical data were expressed as frequency and percentage, and the Chi-square test or Fischer’s exact test was used to compare the groups. Univariate and multivariable logistic regression models were applied to evaluate the effect of the parameters (age, sex, comorbidities, antiviral therapy, severity of symptoms, time from the first test to the first negative test, side effect of antiviral therapy, number of comorbidities, discontinuation of therapy after medical decision, suspension of therapy by voluntary decision, COVID-19 vaccination, days after last vaccination, and days from the onset of symptoms to prescription of antiviral therapy) on the probability of being hospitalized and/or death at 30 days. Using the *p*-values criterion (*p* < 0.25), a stepwise selection was used to estimate the final model. The results of the logistic models are expressed by the Odds Ratios (OR), their 95% Confidence Interval (95% CI), and the *p*-values of the Wald’s tests. Thirty-day progression-free survival toward the composite endpoint (PFSCE) was defined as the time interval between the date of positivity to COVID-19 and the date of hospitalization and/or death within 30 days. Univariate and multivariable Cox-regression models were performed to define associations between 30-day PFSCE and the other parameters. The proportional hazard assumptions for the Cox model were checked, and the results were expressed as hazard ratios (HR) and their 95% Confidence Interval. The survival curves of someone with the significant parameters were drawn with the Kaplan–Meier method.

A *p*-value < 0.05 was considered statistically significant. Statistical analyses were performed by using the SAS/STAT^®^ Statistics version 9.4 (SAS Institute, Cary, NC, USA).

## 3. Results

A total of 719 individuals (51.7% female) were included during the study phase. Among them, 554 (77%) received Molnupiravir, whereas 165 (23%) were NMV-r(NVM) users. The baseline characteristics of the patients are summarized in Table 1. The median age was 71 years (interquartile range, IQR, 61–80). All subjects were Caucasian.

Compared with NMV-r users, subjects receiving Molnupiravir were older (median age 73 vs. 61, *p* < 0.0001), had more comorbidities (*p* = 0.001), suffered mostly from cardiovascular diseases (*p* < 0.001), chronic renal failure with eGFR ≥ 30 mL/min/1.73 m^2^ (*p* = 0.005), and were more likely to take anticoagulants (*p* < 0.001) and antipsychotics/antidepressants (*p* = 0.008).

Overall, 669 (93%) individuals out of 719 had received complete vaccination: 581 (86.8%) received BNT162b2 (Comirnaty), 73 (11%) Moderna m-RNA vaccine, and 15 (2.2%) AstraZeneca. At least one booster dose was administered in 89.1% of patients (89% Comirnaty and 11% Moderna). A total of 31 subjects received a fourth dose.

### 3.1. Safety Profile

Oral antivirals were safe and well-tolerated. The safety profile, according to the antiviral therapy, is reported in Table 2. During antiviral therapy, 85 (11.8%) individuals experienced at least one adverse event. Compared with Molnupiravir users, those receiving NMV-r were more likely to have a bitter mouth (*p* < 0.001), dysgeusia (*p* < 0.001), nausea (*p* = 0.001), and epigastric burning (*p* = 0.02).

Only three serious adverse events (SAEs) were reported: extensive rash in an 80-year-old man taking Molnupiravir, whilst, among the NMV-r users, reversible bradycardia in a 70-year-old woman and an extensive rash in a 57-year-old man.

Treatment discontinuation occurred in 30 individuals (4.1%): 19 (63%) by voluntary decision and 11 (37%) by medical decision. Among the latter, seven subjects developed adverse events, including the three SAEs described above, whereas four individuals, who required hospitalization for pneumonia and oxygen therapy, replaced the oral antiviral treatment with the antiviral Remdesivir.

### 3.2. Clinical Outcomes at 30 Days

Overall, 47 (6.5%) individuals were hospitalized and/or died at 30 days. They formed Group A. Clinical characteristics among the outpatients with (Group A) or without (Group B) composite outcomes are described in Table 3. Subjects in Group A were more likely to have an age ≥ 75 (59.57% vs. 38.24%, *p* = 0.005), a chronic renal failure (21.28% vs. 9.25%, *p* = 0.019), and discontinuation of antiviral treatment after a medical decision (8.51% vs. 1.05%, *p* = 0.004). No significant differences between the two antivirals were observed in terms of the composite outcome.

A total of 43 (5.9%) hospitalizations at 30 days were reported; 20 out of 43 were caused directly by COVID-19 pneumonia, requiring oxygen support, whereas the remaining 23 included 4 to heart failure; 1 to abdominal pain; 2 to surgical interventions; 3 to social reasons; 1 to a stroke; and 12 to the expiry of the general conditions, including severe dehydration, senile cachexia, and feeding difficulties. All-cause hospitalization was associated with age ≥ 75 (OR 2.73; 95% CI 1.445–5.172; *p* = 0.002), moderate illness (OR 14.13; 95% CI 6.258–31.902; *p* < 0.001), chronic renal failure (OR 2.57; 95% CI 1.178–5.596; *p* = 0.010), and a discontinuation in antiviral treatment after medical decision (OR 6.24; 95% CI 1.593–24.409; *p* = 0.008) (Supplementary Table S1).

NMR/r users had a shorter median time to negative test compared with Molnupiravir users (9 days vs. 12 days, *p* < 0.0001). The time from the first positive test to viral clearance was not associated with the composite outcome.

Thirteen deaths were observed: seven due to acute respiratory failure related to COVID-19, three because of advanced malignancies, one for an acute myocardial infarction, and two because of senile cachexia. Among the individuals who died, ten were Molnupiravir users, and two received NMV-r, including a 63-year-old woman suffering from breast cancer with multiple metastases and a 73-year-old man with acute myocardial infarction (Supplementary Table S2).

### 3.3. Factors Associated with the Composite Outcome

As shown in Table 4, a logistic regression model was performed to assess factors associated with the composite outcome. At the univariate analysis, male sex (OR 1.79; 95% CI 0.977–3.292; *p* = 0.050), age ≥ 75 (OR 2.38; 95% CI 1.302–4.349; *p* = 0.005), moderate illness at time of prescription (OR 12.44; 95% CI 5.557–27.84; *p* < 0.001), treatment discontinuation after medical decision (OR 8.79; 95% CI 2.479–31.221; *p* = 0.001), and a greater number of comorbidities (OR 1.51; 95% CI 1.084–2.111; *p* = 0.010) were associated with all-cause hospitalization and/or death at 30 days.

In multivariate analysis, after adjusting for age and sex, male sex (OR 3.78; 95% CI 1.622–8.836; *p* = 0.002), age ≥75 (OR 2.65; 95% CI 1.245–5.628; *p* = 0.012), moderate illness (OR 16.75; 95% CI 6.17–45.48; *p* < 0.001), and treatment discontinuation after medical decision (OR 8.15; 95% CI 1.577–42.114; *p* = 0.012) remained independently associated with the composite outcome. In terms of the composite outcome, no differences between the two antiviral regimens were observed.

### 3.4. Factors Associated with 30-Day Progression-Free Survival toward Composite Endpoint

As shown in Table 5, a Cox regression model was performed to assess factors associated with a 30-day progression-free survival composite endpoint (PFSCE). At univariate analysis, an age over 75 years (HR 2.80; 95%CI 1.426–5.821; *p* = 0.003), moderate illness at time of prescription (HR 13.83; 94%CI 6.727–28.451; *p* < 0.001), treatment discontinuation after medical decision (HR 10.19; 95% CI 3.585–28.972; *p* < 0.001), and a greater number of comorbidities (HR 1.69; 95%CI 1.193–2.389; *p* = 0.003) were associated with 30-day PFSCE. In the multivariate analysis, after adjusting for age and sex, age ≥ 75 (HR 2.76; 95%CI 1.339–5.672; *p* = 0.005), moderate illness (HR 10.97; 95%CI 5.184–23.207; *p* < 0.001), treatment discontinuation after medical decision (HR 9.42; 95%CI 3.157–28.1; *p* < 0.001), and number of comorbidities (HR 1.67; 95%CI 1.178–2.379; *p* = 0.004) were associated with 30-day PFSCE.

**Figure 1:**
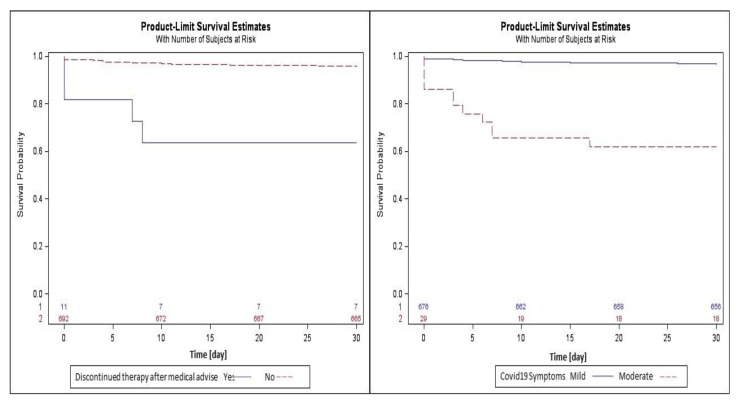
Survival curves drawn with the Kaplan–Meier method for the parameter COVID-19 symptoms and discontinuation of therapy for medical advice.

Figure 1 shows the survival curves drawn with the Kaplan–Meier method. In the curve stratified by COVID-19 symptoms, the mean (±standard error) of survival of patients with moderate symptoms was less compared with the patients with mild symptoms (12.17 ± 1.33 vs. 29.30 ± 0.16). Patients who discontinued antiviral therapy after a medical decision survived less often than those who continued (6.45 ± 1.06 vs. 29.06 ± 0.18).

## 4. Discussion

SARS-CoV2 treatment with antivirals has recently been enriched by the introduction of Molnupiravir and NMV-r. Although a short three-day Remdesivir treatment was added to prevent severe COVID-19 [19], oral antivirals have undoubted advantages in limiting organizational issues and healthcare costs [4,11]. NMV-r is considered by the current international guidelines to be the first-line antiviral treatment and has obtained approval by EMA [18]. Molnupiravir, on the other hand, should be prescribed only when the use of NMV-r is contraindicated.

Furthermore, since new monoclonal antibodies that are effective against circulating variants are not to be immediately available [7,20], in many countries, including Italy, oral antivirals along with Remdesivir are currently the effective treatments to be used to stem severe COVID-19 in high-risk individuals [21]. In addition, anti-SARS-CoV-2 antivirals may be useful in reducing the symptoms of COVID-19 that can compromise the quality of life among the individuals with a recent infection [3].

We reported our real-life experience with oral antivirals in a setting of high-risk outpatients in a period where numerous variants of concern were circulating, including Omicron and its subvariants. Additionally, a comparison of the two oral antivirals in terms of effectiveness and safety profile was performed.

Randomized trials of NMV-r and Molnupiravir demonstrated a reduction in the risk of progression to severe COVID-19 that was 89% and 31%, respectively, lower than placebo groups [14,15]. Of note, MOVe-OUT and EPIC-HR trials were conducted in young and unvaccinated individuals.

Differently, our study evaluated older subjects (median age of 71 years) who were either fully vaccinated (93%) or received at least one booster dose (89%).

Two recent real-life studies evaluated the efficacy of NMV-r and Molnupiravir. In both studies, the authors used a propensity-score-matched analysis [22,23]. In particular, in the NMV-r study, the authors demonstrated a reduced risk of composite outcome among NMV-r users compared with non-users (7.8% vs. 14.4%), thus resulting in a 45% relative risk reduction [22]; in the Molnupiravir study, a non-significantly reduced risk of the composite outcome was evidenced. However, the authors reported a significant decrease in the risk of disease progression in specific subgroups, such as older patients, females, and patients with inadequate COVID-19 vaccination [23].

Another real-life study reported a rapid improvement in COVID-19 symptoms at phone follow-up 5 days after the initial evaluation and initiation of Molnupiravir [24].

In our study, a composite outcome occurred in 47 individuals (6.5%), similar to that reported in the Ganatra et al. study. However, concerning this study, our data included both NMV-r users and Molnupiravir users, without an untreated group. We did not observe significant differences among the two antivirals in the composite outcome (6.8% in Molnupiravir users vs. 5.4% in NMV-r users), even among the 50 (7%) unvaccinated patients. The lack of difference between the two regimes might be explained by several reasons. Primarily, the two groups had such differences as the older age in the Molnupiravir group and the higher percentage of active malignancies in the NMV-r group. With both of these categories being at high risk of hospitalization, the composite outcome could be balanced. Another reason might be the smaller number of patients in the NMV-r group. Furthermore, although the median time since the last vaccine dose was not particularly short (median, 132 days), its protective effect in preventing severe COVID-19 was likely to be maintained, thus helping to limit hospitalizations and deaths. Finally, some evidence suggests that the clinical features of Omicron and subvariants, although more likely to evade the vaccine, may be milder than those caused by the alpha and the delta variants [25].

Although viral clearance was not an endpoint of our study, we found that NMR/r users had a shorter time to negative test compared with Molnupiravir users (9 days vs. 12 days, *p* < 0.0001). However, numerous biases should be considered. First, Molnupiravir users were older subjects, sometimes bedridden at home, and with a greater number of comorbidities. Thus, the amount of time from the first positive test to viral clearance could be longer in this group, partly due to the difficulties of carrying out the test. Second, the types of COVID-19 tests (antigenic or molecular), as well as the timing of performing the swab to ascertain healing, could differ. At any rate, the time to viral clearance was not associated with the composite outcome.

We decided to include in the analysis all the causes of hospitalization and death, as we believe that COVID-19 disease can cause direct (interstitial pneumonia and acute respiratory failure) and indirect consequences, including increased risk of cardiovascular events, heart exacerbation, respiratory and renal diseases, risk of falling, and difficulty in hydration and feeding, particularly among the elderly and frail individuals [26,27].

Male sex, age ≥ 75 years, moderate illness at the time of prescription, and treatment discontinuation after medical decision were factors independently associated with the composite outcome. Furthermore, the discontinuation of treatment after a medical decision deserves an explanation, unlike the first three factors that have been largely associated with severe COVID-19 in several studies [28,29,30,31]. We reported eleven discontinuations due to medical advice determined by side effects in seven cases without hospitalizations/or deaths at 30 days. The remaining four were due to hospitalizations, including two subjects with advanced malignancies who stopped the oral antiviral, received Remdesivir after developing respiratory failure, and subsequently died. Thus, we assume that antiviral discontinuation due to medical advice might be an effect of hospitalization rather than a cause. In our study, 19 subjects voluntarily stopped the treatment. Possible reasons included the lack of need for antiviral therapy due to subjective clinical improvement and the fear of developing side effects in the course of treatment.

Both antivirals were well tolerated, and serious adverse events leading to treatment discontinuation were rare (0.4%). Most of the self-reported side effects were mild and could be partly attributable to the disease itself. Interestingly, they were more frequent in NMV-r users than in those receiving Molnupiravir. A possible explanation may be that NMV-r users were overall younger and consequently were able to report in more detail the effects experienced during treatment. Another reason may be the presence of Ritonavir, which is notoriously associated with the reported side effects, including dysgeusia, diarrhea, and nausea. In addition, since Ritonavir is a CYP3A4 inhibitor, it also involves interactions and alterations in the efficacy of numerous other drugs; this makes managing their co-administration somewhat difficult [17,32].

Moreover, a careful drug–drug interactions evaluation was essential for choosing the most appropriate antiviral therapy. Consequently, for many frail subjects who took life-saving drugs, such as anticoagulants, antiarrhythmics, or immunosuppressants, the choice of antiviral treatment was directed toward Molnupiravir, which, unlike NMV-r, has a good profile of drug–drug interactions and does not require dose adjustments based on renal filtrate.

Our study population, mainly consisting of elderly and vulnerable subjects, was treated predominantly with Molnupiravir (77%). Molnupiravir was more frequently prescribed partly for the above reasons and partly because of the current therapeutic choices in Italy (as reported in the AIFA monitoring records) and the unavailability of NMV-r in January and February 2022. In addition, from May onward, NMV-r could be directly prescribed by GPs who have to compile a treatment plan.

Our study presents several limitations. Firstly, this is a single-center retrospective observational study. Secondly, we did not have a matched control group including untreated subjects. Third, since the study population assessed only outpatients, biochemical and radiological data during the illness were unavailable, except for hospitalized individuals. A further limitation of our study is that we cannot provide clear information if any COVID-19 infections had occurred in the past.

To our knowledge, this is the first European study comparing the two regimens in terms of safety profile and efficacy from a real-life setting. Furthermore, since the role of oral antivirals in preventing severe COVID-19 among fully vaccinated high-risk subjects is still under investigation, our observations provide valuable insights from clinical practice.

Notwithstanding, we did not observe any differences between the two antivirals in terms of clinical outcomes consistent with recent studies; we reported that older people with multiple comorbidities were more likely to be hospitalized and/or die at 30 days compared with the younger, regardless of vaccination status. Thus, a timely antiviral treatment in these categories should be prioritized and pursued as soon as possible. Finally, in high-risk and fragile individuals with impaired response to COVID-19 vaccination, a series of measures might be envisaged, including periodic booster doses, the use of effective monoclonal antibodies, and a prolonged antiviral therapy, as well as a combination of antivirals to consider in future research goals.

## 5. Conclusions

Early use of oral antivirals may limit hospital admissions, reduce COVID-19-related morbidity or mortality, and reduce healthcare costs. Therefore, it also becomes necessary to increase efforts to establish and strengthen a network between COVID-19 referral hubs and GPs in order to to identify individuals who may benefit from these therapies.

## Figures and Tables

**Table 1:** Clinical characteristics according to antiviral therapy.

	Total (N = 719)	Molnupiravir (N = 554)	Nirmatrelvir/r (N = 165)	*p*-Value
Age	71 (61–80)	73 (64–82)	62 (50.5–73)	<0.001
Sex				
Female	372 (51.7)	280 (50.5)	92 (55.7)	0.240
Comorbidities				
Cardiovascular diseases	313 (43.5)	285 (51.4)	28 (16.8)	<0.001
Diabetes mellitus	125 (17.3)	101 (18.2)	24 (14.4)	0.270
Obesity, BMI ≥ 30	164 (22.8)	122 (22.1)	42 (25.3)	0.350
Malignancies	134 (18.6)	90 (16.2)	44 (26.5)	0.002
Respiratory diseases	145 (20.1)	110 (19.8)	35 (21.1)	0.700
Chronic renal failure, eGFR ≥ 30 mL/min	72 (10)	65 (11.7)	7 (4.2)	0.005
Immunodeficiency primary or secondary	105 (14.6)	73 (13.1)	32 (19.2)	0.040
Nervous system diseases	29 (4)	22 (3.9)	7 (4.2)	0.870
Two or more comorbidities	269 (37.4)	225 (40.6)	44 (26.5)	0.001
Mild COVID-19	689 (95.9)	529 (95.5)	160 (96.3)	0.400
Moderate COVID-19	30 (4.1)	25 (4.5)	5 (3.7)	0.400
Days from onset of symptoms to antiviral prescription	2 (2–3)	2 (2–3)	3 (2–3)	0.300
Guests of long-term facilities	30 (4.1)	30 (5.4)	0	0.007
Full vaccination	669 (93)	518 (93.5)	151 (90.9)	0.370
Patients who underwent booster to anti-SARS-CoV2 vaccine	641 (89.1)	495 (89.3)	146(87.9)	0.750
Time (days) from last dose of vaccine to positive swab	135 (106–165)	135 (106–165)	135 (106–135)	0.400
Complete data available on main comedications	276	210	66	
Anticoagulants	55 (19.9)	55 (26.2)	0	<0.001
Statins	62 (22.4)	51 (24.2)	11 (16.6)	0.190
Beta blockers	32 (11.5)	28 (13.3)	4 (6)	0.100
Calcium antagonists	28 (10.1)	23 (10.9)	5 (7.5)	0.420
Bronchodilators	22 (7.9)	16 (7.6)	6 (9.1)	0.700
Sartans	27 (9.7)	21 (10)	6 (9.1)	0.820
Ace-inhibitors	17 (6.1)	13 (6.2)	4 (6)	0.960
Antiplatelet agents	50 (18.1)	35 (16.6)	15 (22.2)	0.260
Pump protonic inhibitors	28 (10.1)	25 (11.9)	3 (4.5)	0.080
Antiepileptic drugs	12 (4.3)	10 (4.7)	2 (3)	0.540
Antipsychotics/Antidepressants	34 (12.3)	32 (15.2)	2 (3)	0.008
Immunosuppressants	20 (7.2)	14 (6.6)	6 (9.1)	0.500
Antidiabetic drugs	58 (21)	48 (22.8)	10 (15.1)	0.180
Anticancer drugs	22 (7.9)	16 (7.6)	6 (9.1)	0.700
Data are shown as median (IQR) or number (%)				

**Table 2:** Safety profile and outcome according to the antiviral therapy.

	Molnupiravir (N = 554)		Nirmatrelvir (N = 165)		
Side Effects	N	%	N	%	*p*-Value
Bitter mouth	2	0.37%	20	12.66%	<0.001
Dysgeusia	0	0.00%	9	5.70%	<0.001
Headache	5	0.92%	2	1.27%	0.659
Diarrhea	17	3.14%	8	5.06%	0.327
Fatigue	5	0.92%	0	0.00%	0.593
Nausea	11	2.03%	14	8.86%	0.001
Itching	1	0.18%	0	0.00%	1.000
Rash	2	0.37%	3	1.90%	0.079
Xerostomia	1	0.18%	0	0.00%	1000
Dizziness	3	0.55%	0	0.00%	1000
Epigastric burning	5	0.92%	6	3.80%	0.026
Muscular pains	2	0.37%	0	0.00%	1.000
Serious adverse events	1	0.18%	2	1.22%	0.133
Discontinuation of therapy					
Voluntary	14	2.54%	5	3.05%	0.781
Medical decision	4	0.72%	7	4.27%	0.004
Outcome					
Hospitalization	36	6.50%	7	4.24%	0.351
Death	11	1.99%	2	1.21%	0.742

**Table 3:** Clinical characteristics according to the composite outcome (hospitalization and/or death at 30 days).

	Patients with Composite Outcome (Group A)		Patients without the Composite Outcome (Group B) (N = 672)		*p*-Value
	(N = 47)				
	N	%	N	%	
Sex					
Male	29	61.70%	318	47.32%	0.070
Age ≥ 75	28	59.57%	257	38.24%	0.005
Comorbidities					
Cardiovascular diseases	24	51.06%	289	43.13%	0.292
Chronic pulmonary diseases	14	29.79%	131	19.55%	0.094
Immunodeficiency	6	12.77%	99	14.78%	0.833
Diabetes	5	10.64%	120	17.91%	0.238
Obesity					
Chronic renal failure, eGFR ≥ 30 mL/min	10	21.28%	62	9.25%	0.019
Malignancies	13	27.66%	121	18.06%	0.120
Neurological diseases	4	8.51%	25	3.73%	0.114
≥2 comorbidities	21	44.68%	248	36.96%	0.350
Antiviral Therapy					
Molnupiravir	38	80.85%	516	76.79%	0.594
Nirmatrelvir	9	19.15%	156	23.21%	
Prescription within 48 h	21	44.68%	351	52.23%	0.366
Discontinuation of therapy					
Voluntary	3	6.38%	16	2.39%	0.123
Medical advice	4	8.51%	7	1.05%	0.004
Al least one side effect	3	6.67%	81	12.39%	0.345
Serious adverse events	0	0,00%	3	0.45%	1.000
Vaccination status					
First complete vaccination course	43	91.49%	626	93.15%	0.560
Booster	40	85.11%	601	89.43%	0.334

**Table 4:** Factors associated with the composite outcome (all-cause hospitalization and or death at 30 days).

	Univariate Model			Multivariate Model		
Parameter	OR ^1^	IC95%	*p*-Value	OR ^1^	IC95%	*p*-Value
Sex (male vs. female)	1.79	0.977–3.292	0.059	3.79	1.622–8.836	0.002
Age ≥75 (yes vs. no)	2.38	1.302–4.349	0.004	2.65	1.245–5.628	0.012
Molnupiravir vs. NMV-r (yes vs. no)	1.27	0.604–2.697	0.522			
Prescription within 48 h (yes vs. no)	0.74	0.408–1.339	0.318			
Moderate vs. mild COVID-19	12.44	5.557–27.84	<0.001	16.75	6.17–45.485	<0.001
Full cycle of Vaccination (yes vs. no)	0.79	0.272–2.296	0.664			
At least one booster dose (yes vs. no)	0.67	0.291–1.563	0.359			
Side effects (yes vs. no)	0.50	0.153–1.668	0.263			
Voluntary discontinuation (yes vs. no)	2.78	0.781–9.912	0.114			
Medical discontinuation (yes vs. no)	8.79	2.479–31.221	0.001	8.15	1.577–42.114	0.012
Number comorbidities (+1 disease)	1.51	1.084–2.111	0.014			
Time from the first positive test to the first negative (1 day)	1.01	0.996–1.018	0.204			
Days from the onset of symptoms to antiviral treatment (1 day)	1.10	0.827–1.451	0.524			
Days from the last vaccination to the first positive test (1 day)	1.00	0.996–1.006	0.751			

^1^ Adjusted by Wald methods; IC, Confidence Interval; OR, Odds Ratio.

**Table 5:** Factors associated with 30-day progression-free survival toward composite endpoint.

	Univariate Model			Multivariate Model		
Parameter	HR ^1^	IC95%	*p*-Value	HR ^1^	IC95%	*p*-Value
Sex (male vs. female)	1.24	0.634–2.436	0.527	0.66	0.335–1.318	0.242
Age ≥75 (yes vs. no)	2.80	1.426–5.821	0.003	2.76	1.339–5.672	0.005
Antiviral (Molnupiravir vs Nirmatrelvir)	1.16	0.504–2.656	0.731			
Prescr within 48 h (yes vs. no)	0.92	0.47–1.804	0.809			
Moderate COVID-19 (yes vs. mild)	13.83	6.727–28.451	<0.001	10.97	5.184–23.207	<0.001
Full vaccination (yes vs. no)	0.57	0.2–1.608	0.285			
Booster (yes vs. no)	0.69	0.269–1.792	0.450			
Side effects (yes vs. no)	0.11	0.007–1.915	0.130			
Voluntary discontinuation (yes vs. no)	2.44	0.585–10.181	0.221			
Medical discontinuation (yes vs. no)	10.19	3.585–28.972	<0.001	9.42	3.157–28.1	<0.001
Number comorbidities (+1 disease)	1.69	1.193–2.389	0.003	1.67	1.178–2.379	0.004
Time to first negative swab (+1 day)	1.01	0.997–1.015	0.194			
Time to prescription (+1 day)	0.99	0.712–1.369	0.939			
Days from last vaccination dose (+1 day)	1.00	0.997–1.009	0.387			

IC, Confidence Interval; HR, Hazard Ratio. ^1^ Adjusted by Wald methods.

## Data Availability

The data that support the findings of this study are available from the corresponding author upon reasonable request.

## Supplementary Materials

The following are available online at https://www.mdpi.com/article/10.3390/v14112514/s1, Table S1: Factors associated with all-cause hospitalization, Table S2: Factors associated with death.
