# Supplementary material for: Early Access to Oral Antivirals in High-Risk Outpatients: Good Weapons to Fight COVID-19
Source: Viruses. 2022 Nov 14;14(11):2514. doi: 10.3390/v14112514 (PMC9695104; doi:10.3390/v14112514)
Supplement: Supplementary file 1 [file viruses-14-02514-s001.zip › viruses-1986503-supplementary.pdf]

Table S1. Factors associated with all-cause hospitalization

**Outcome: Hospitalization**	*Univariate Model*		
**Parameter**	**OR[1]**	**IC95%**	**p-value**
Sex [male vs female]	1.877	0.993 - 3.548	0.0524
Age ≥75 [Yes vs No]	2.734	1.445 - 5.172	0.002
Therapy [Molnupiravir vs Nirmatrelvir]	1.569	0.685 - 3.594	0.2871
prescr 48h [Yes vs No]	0.724	0.389 - 1.347	0.3085
Moderate vs mild COVID19	14.129	6.258 - 31.902	<0.0001
Full vaccination [Yes vs No]	0.996	0.297 - 3.341	0.9952
Booster [Yes vs No]	0.735	0.3 - 1.801	0.5003
Side effects [Yes vs No]	0.562	0.17 - 1.864	0.3464
Voluntary suspension [Yes vs No]	1.882	0.421 - 8.426	0.4081
Discontinuation after medical advice [Yes vs No]	6.238	1.593 - 24.409	0.0086
number comorbidities [+1 disease]	1.418	0.996 - 2.019	0.0528
2+ comorb [Yes vs No]	1.216	0.651 - 2.274	0.5396
Cardiovascular diseases [Yes vs No]	1.523	0.821 - 2.826	0.1824
Respiratory diseases [Yes vs No]	1.779	0.903 - 3.505	0.0958
immunodeficiency [Yes vs No]	0.942	0.387 - 2.29	0.8949
diabetes [Yes vs No]	0.469	0.164 - 1.336	0.1564
obesity [Yes vs No]	0.426	0.165 - 1.01	0.0783
Chronic renal failure [Yes vs No]	2.567	1.178 - 5.596	0.0177
Malingancy [Yes vs No]	1.54	0.755 - 3.14	0.2349
Neurological diseases [Yes vs No]	1.872	0.543 - 6.45	0.3234
time to negative test [1 day]	1.007	0.997 - 1.018	0.1882
time to prescr [1 day]	1.109	0.827 - 1.485	0.4901
day_vacc [1 day]	1.001	0.996 - 1.007	0.6623

[1] adjusted by Wald methods; IC: Confidence interval; OR: Odds Ratio

Table S2. Factors associated with Death

**Outcome: Death**	***Univariate Model***		
**Parameter**	**OR[1]**	**IC95%**	**p-value**
Sex [male vs female]	0.47	0.144 – 1.541	0.213
Age ≥ 75 [Yes vs No]	2.478	0.802 – 7.652	0.1147
Antiviral [Molnupiravir vs Nirmatrelvir]	1.651	0.362 – 7.524	0.5171
prescr 48h [Yes vs No]	1.503	0.487 – 4.639	0.4785
Moderate COVID-19 [yes vs no]	24.328	7.597 – 77.907	<0.0001
Full vaccination [Yes vs No]	0.401	0.86 – 1.862	0.2435
booster [Yes vs No]	0.663	0.144 – 3.05	0.5981
Side effects [Yes vs No]	0.605	0.078 – 4.716	0.6319
Voluntary discontinuation [Yes vs No]	3.712	0.391 – 25.717	0.2797
Discontinuation after medical decision [Yes vs No]	14.02	2.709 – 72.557	0.0016
number comorbidities [+1 disease]	1.621	0.906 – 2.901	0.104
2+ comorb [Yes vs No]	1.441	0.479 – 4.333	0.5156
Cardiovascular diseases [Yes vs No]	0.381	0.104 – 1.397	0.1457
Respiratory diseases [Yes vs No]	0.714	0.156 – 3.255	0.6631
immunodeficiency [Yes vs No]	0.481	0.062 – 3.737	0.484
diabetes [Yes vs No]	0.859	0.188 – 3.923	0.844
obesity [Yes vs No]	0.608	0.133 – 2.772	0.5207
Chronic renal failure [Yes vs No]	4.158	1.248 – 13.861	0.0203
Malignancy [Yes vs No]	7.934	2.362 – 22.809	0.0006
Neurological diseases [Yes vs No]	4.566	0.964 – 21.619	0.0556
time to neg [+1 day]	0.988	0.865 – 1.129	0.8575
time to prescr [+1 day]	0.791	0.455 – 1.375	0.4057
day_vacc [+1 day]	0.996	0.985 – 1.007	0.4577

[1] adjusted by Wald methods; IC: Confidence interval; OR: Odds Ratio
